# Fixation of Olecranon Fractures Using a Hybrid Intramedullary Screw and Tension Band Construct

**DOI:** 10.1155/2024/6471544

**Published:** 2024-05-30

**Authors:** Kaitlin Rush, John Fisher, Neil Jain, Caleb Gottlich, Cyrus Caroom

**Affiliations:** Department of Orthopedic Surgery, Texas Tech University Health Sciences Center, Lubbock, Texas, USA

## Abstract

**Introduction:**

Olecranon fractures are common injuries that require surgical intervention for optimal outcomes. Various fixation methods have been described in the literature, including the use of intramedullary proximal ulna screws in combination with tension band augmentation. Limited research has compared this hybrid technique to other established methods of fixation. This study compared complication and reoperation rates between multiple groups.

**Methods:**

A retrospective review was conducted on patients with olecranon fractures who underwent internal fixation at a level 1 trauma center between January 1st, 2013, and April 22nd, 2023. Data was collected using CPT codes, and patients were categorized into five groups based on the method of fixation received: no implant, tension band only, locking olecranon plate, intramedullary screw and tension band hybrid, and others. Variables such as patient demographics, Mayo fracture classification, open vs. closed injury, implant type, reoperation rates, and postoperative complications were recorded.

**Results:**

A total of 217 patients were included in the study. No difference was found with implant choice and reoperation rate (*p* = 0.461). There was a significant difference found with reoperation and fracture type (*p* = 0.027) and open fracture (*p* = 0.002).

**Conclusion:**

The primary findings of this study indicate no significant difference in implant choice and reoperation rates among the various fixation methods used for olecranon fractures. These findings suggest that the hybrid fixation technique, utilizing intramedullary proximal ulna screws in combination with tension band augmentation, is a viable and comparable treatment option when evaluated against other well-documented methods of fixation. This study also reiterates that severity of initial injury is often the most important factor related to poorer outcomes. Further discussion and analysis of the data will provide a comprehensive understanding of implications and recommendations for olecranon fracture fixation.

## 1. Introduction

Olecranon fractures account for approximately 10% of all upper extremity fractures in adults and are most commonly caused by direct trauma to the olecranon or an indirect pulling of the triceps on a pronated forearm [1–8]. Although there is a role for nonoperative management in certain patient populations, the majority of these injuries undergo operative fixation [1–15]. Surgical repair of the proximal ulna poses a unique challenge due to the intraarticular nature of most fractures and the scant soft tissue envelope covering the bony surface [3]. Inadequate fixation can result in complications such as chronic pain, posttraumatic arthrosis, contracture, or progressive joint degeneration due to persistent elbow instability [1–3, 6].

**Figure 1:**
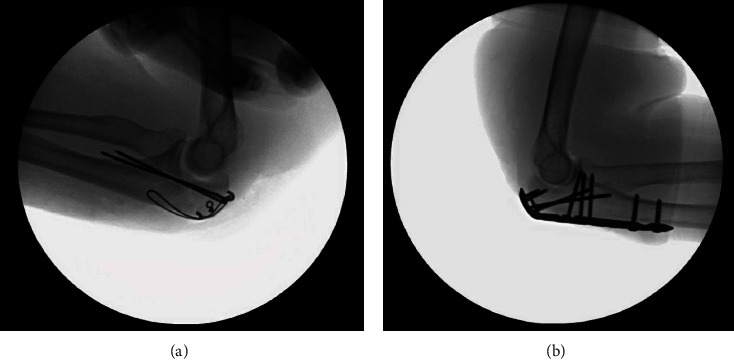
(a) Olecranon fracture repair with a tension band wire construct; (b) olecranon fracture repair with a locking plate/screw fixation.

Among many schemas for describing olecranon fractures, the Mayo classification is a simple and frequently used classification system that includes fracture morphology and stability [1–3, 7, 9]. Mayo type 2A fractures account for 85% of injuries and are most commonly treated with either tension band wiring (TBW) or locking plate/screw fixation (PF) (Figure 1) [1–15]. Both techniques have demonstrated good union rates, postoperative functional scores, and patient-rated outcomes in simple olecranon fractures [1, 2, 4–6, 8, 10–14]. However, hardware prominence and irritation are well-known complications of these operative methods [1, 2, 4–8, 10–13, 15]. TBW fixation more frequently requires symptomatic removal of hardware caused in part by proximal migration of Kirschner wires used for these constructs [1–7, 10–13]. In comparison, PF is estimated to cost 2.6 times more than TBW, even when accounting for higher reoperation rates in the latter [4, 6, 10, 12].

**Figure 2:**
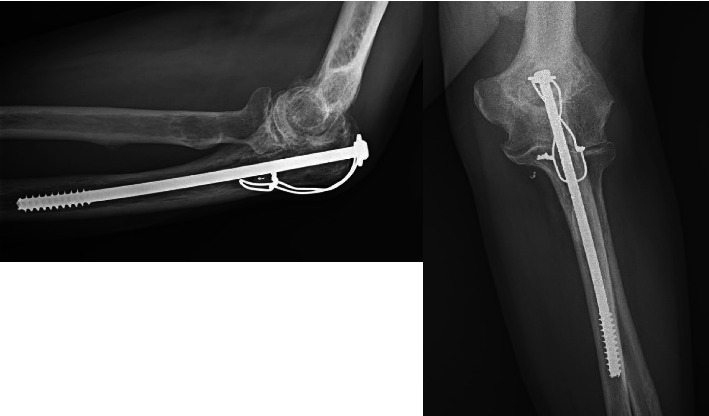
The hybrid construct used in this study consisted of an intramedullary proximal ulna screw and tension band construct.

In addition to the previously discussed techniques, intramedullary screws have been described as a fixation construct for olecranon fractures, either alone or in hybrid constructs with plate or tension band augmentation (Figure 2) [7, 8, 13–15]. Biomechanical studies have demonstrated that intramedullary proximal ulna screws in combination with tension band augmentation provide superior fixation when compared to TBW or PF alone [7, 14]. Although this hybrid technique has been described previously, there is a limited amount of data comparing hybrid intramedullary screw constructs to isolated TBW and PF constructs [8, 15].

The purpose of this study was to fill a gap in evidence comparing hybrid intramedullary screw/tension band fixation to the frequently described standards of care. Although TBW alone has a high rate of prominent hardware irritation, fixing the wire in place beneath a large diameter cortical screw and washer may prevent proximal migration and soft tissue irritation. This study compared complication and reoperation rates between multiple constructs used for simple olecranon fracture fixation.

## 2. Materials and Methods

A retrospective chart review was conducted on patients with olecranon fractures who underwent internal fixation at a single level 1 trauma center between January 1st, 2013, and April 22nd, 2023. Data from the electronic medical record were collected using CPT codes, and patients were categorized into five groups based on the method of fixation received: no implant, tension band only, locking olecranon plate, intramedullary screw and tension band hybrid, and others. The “others” classification was given to fixations not fitting into previous categories. This included other hybrid constructs (i.e., cannulated screw with adjunct plate fixation), or an intramedullary screw alone. Specific exclusion criteria included those less than 18 years of age or skeletally immature patients.

Variables such as patient demographics, Mayo fracture classification, open versus closed injury, implant type, reoperation rates, and postoperative complications were recorded. Secondary variables included diabetes and smoking status, operative time, and estimated blood loss. Statistical analysis was performed using IBM SPSS Statistics software (version 29.0, IBM Corp., Armonk, NY, USA).

### 2.1. Operative Technique

Patients are placed under general endotracheal anesthesia per the anesthesia providers. All patients are positioned in lateral decubitus with the operative extremity up. A radiolucent arm board is connected to the operating table anterior to the patient's chest/abdomen. The operative arm is draped over the arm board to rest at approximately 90°. C-arm fluoroscopy is then able to approach parallel to the operating table to obtain imaging. The operative extremity is prepped and draped.

A direct longitudinal approach to the olecranon is performed. Fracture is identified, debrided, and reduced. The reduction tool of choice is pointed reduction clamps. A guidewire is then inserted directly through the tip of the olecranon and into the medullar canal of the ulna to facilitate a 5.0 cannulated drill bit. Either a 6.5 mm or 7.3 mm partially threaded cannulated screw may be used. Screw size is determined by premeasured ulnar medullary canal diameter as well as intraoperative cortical fit.

The selected screw is then inserted with a washer and left approximately 1 cm proud at the tip of the olecranon. A Luque wire is then used to create a tension band construct, placing the midpoint of the wire beneath the washer. A transverse bicortical hole is drilled approximately 3 cm distal to the fracture site. The wire is crossed in a figure-of-eight configuration. A loop is created on either the medial or lateral wire, and the contralateral side is passed through the previously drilled hole. The remaining free wire ends are twisted to tighten the construct while the screw and washer are simultaneously tightened. The free ends of the wire can then be clipped at the ends of the twist, which may be tamped down into the bone to prevent prominence.

## 3. Results

217 patients were included in the study. No difference was found in sex (*p*=0.660), presence of diabetes (*p*=0.594), smoking status (*p*=0.666), and laterality (*p*=0.809) (Table 1).

BMI, OR time, and blood loss were logarithmically transformed to produce parametric data confirmed with Q-Q plots and Shapiro–Wilk testing. These were then tested with one-way ANOVA and Bonferroni correction. There was no difference in BMI between groups (*p*=0.05) (Table 1). OR time and blood loss were found to be higher in the locking plate group (*p* ≤ 0.001). Estimated blood loss was found to be higher in the locking plate group than in the other treatment groups (*p*=0.013). The locking plate group had an OR time significantly higher than the no-implant group (*p* ≤ 0.001) and the other treatment group (*p*=0.003). No difference in OR time was found between the locking plate group and the intramedullary screw plus tension band group (*p*=1.000) or the tension band group (*p*=0.576).

**Figure 3:**
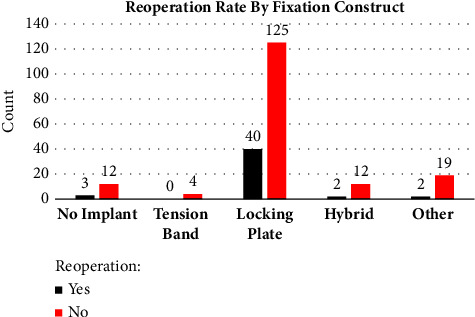
Reoperation rate by fixation construct. No difference was found with implant choice and reoperation rate.

**Figure 4:**
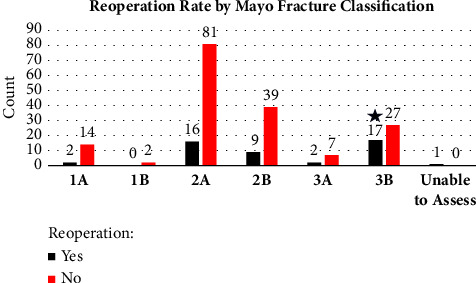
Reoperation rate by Mayo fracture classification. There was a significant difference found with reoperation and fracture type, with increased reoperation rates in increasingly complex fracture patterns (Mayo 3B).

**Figure 5:**
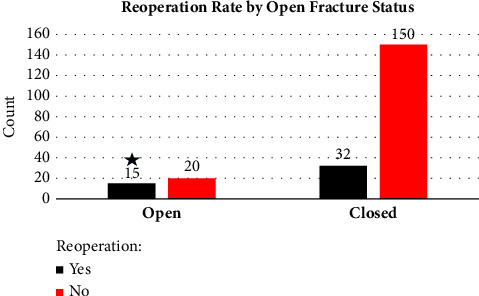
Reoperation rate by open fracture status. There was a significant difference in reoperation rates between open vs. closed fractures, with open fractures having an increased reoperation rate.

Chi-squared tests were used to compare implant choice and our primary outcome, reoperation rate, and secondary outcomes of fracture type and presence of open fracture. No difference was found with implant choice and reoperation rate (*p*=0.461) (Tables 1 and 2) (Figure 3). There was a significant difference found with reoperation and fracture type (*p*=0.027) with increased reoperation rates in increasingly complex fracture patterns (Mayo 3B) (Figure 4). There also was a significant difference in reoperation rates between open vs. closed fractures, with open fractures having an increased reoperation rate (*p* ≤ 0.01) (Figure 5).

## 4. Discussion

The primary findings of this study indicate no significant difference in implant choice and reoperation rates among the various fixation methods used for olecranon fractures. These findings suggest that the hybrid fixation technique, utilizing intramedullary proximal ulna screws in combination with tension band augmentation, is a viable and comparable treatment option when compared to other well-documented methods of fixation.

A typical problem with tension band fixation of olecranon fractures is hardware prominence. The proximity of the hardware to the skin in addition to the scant soft tissue envelope about the elbow creates a difficult working environment for fracture fixation. The goal of hybrid fixation is to provide increased biomechanical stability to a fixation construct while also avoiding postoperative complications and need for reoperation due to hardware prominence and all causes.

The intramedullary screw and tension band construct was implemented at our institution with the idea that fixation of the tension band wire beneath the washer of an intramedullary screw would prevent any proximal migration of the wire and ideally prevent hardware irritation requiring future removal without compromising fracture fixation and healing. Of the fourteen included patients who were treated with hybrid fixation, two have required subsequent hardware removal for soft tissue irritation. All have appropriate radiographic fracture healing with no subsequent malunion, nonunion, or hardware failure recorded.

This study also reiterates that the severity of initial injury is additionally an important factor related to poorer outcomes. In this case, the most complex fracture patterns, as well as open fractures, required repeat surgeries more frequently.

### 4.1. Limitations

This study is a retrospective study; therefore, no functional or subjective patient outcomes are included. The treatment groups are small with unequal distribution of fixation constructs. While we did include the Mayo classification in order to differentiate fracture severity, we did not correlate the fixation construct with the severity of fracture, which could represent a confounding relationship as more complex fracture patterns cannot be appropriately treated with all described fixation constructs.

## 5. Conclusions

The goal of this study was to introduce a seldom-used hybrid fixation construct for simple olecranon fracture patterns. In the appropriate scenario, a hybrid intramedullary screw and tension band construct can be a safe, affordable option with no increase in complications when compared to previously described standards of care.

## Figures and Tables

**Table 1:** Treatment groups and relevant demographics.

	Closed treatment (*n* = 15)	Tension band (*n* = 4)	Locking plate (*n* = 166)	Hybrid (*n* = 14)	Other (*n* = 19)	*p* value
M/F	9/6	1/3	88/78	7/7	12/7	0.66
Diabetic	1	1	27	2	1	0.594
Smoker	1	0	10	0	0	0.666
Laterality (R/L)	6/9	1/3	78/88	6/8	7/12	0.809
BMI	23.88	24.52	27.11	26.95	23.59	0.05
Required reoperation^*∗*^ (#)	3	0	40	2	2	0.461
Operative time (mins)	60.47	68.00	143.76	108.79	88.74	
Open fractures (#)	7	0	24	2	2	
Estimated blood loss (cc)	31	28	99.52	33.21	47.74	

^*∗*^Reasons for reoperation are included in Table 2.

**Table 2:** Reasons for reoperation.

	Closed treatment (*n* = 3)	Tension band (*n* = 0)	Locking plate (*n* = 40)	Hybrid (*n* = 2)	Other (*n* = 2)
Reconstruction of ligaments	1	—	1	—	—
Deep infection	1	—	17	—	—
Additional/revision ORIF	1	—	6	—	—
Prominent/symptomatic implant	—	—	9	2	2
Ulnar nerve neuropathy	—	—	5	—	—
Heterotopic ossification/posttraumatic OA	—	—	2	—	—
Time until reoperation (mean months)	18	—	3.36	10.5	2.5

## Data Availability

Deidentified data gathered during this retrospective review are available from the authors upon reasonable request.
